# Seven Shades of Grey: A Follow-Up Study on the Molecular Basis of Coat Colour in Indicine Grey Cattle Using Genome-Wide SNP Data

**DOI:** 10.3390/genes13091601

**Published:** 2022-09-07

**Authors:** Gabriele Senczuk, Vincenzo Landi, Salvatore Mastrangelo, Christian Persichilli, Fabio Pilla, Elena Ciani

**Affiliations:** 1 Department of Agricultural, Environmental and Food Sciences, University of Molise, 86100 Campobasso, Italy; 2 Department of Veterinary Medicine, University of Bari “Aldo Moro”, 70010 Valenzano, Italy; 3 Department of Agricultural, Food and Forest Science, University of Palermo, 90128 Palermo, Italy; 4 Department of Biosciences Biotechnologies and Biopharmaceutics, University of Bari “Aldo Moro”, 70125 Bari, Italy

**Keywords:** coat colour, cattle, *Bos taurus indicus*, selection signatures, F_st_

## Abstract

Shades of grey and brown are a dominant component in mammal coat colours, representing a fundamental trait involved in a great number of processes including cryptism, sexual selection and signalling. The genetic mechanisms of the grey colouration in mammals are very complex and controlled by hundreds of genes whose effects and interactions are still largely unclear. In this study, we adopted a robust multi-cohort F_st_ outlier approach based on pairwise contrasts between seven grey indicine cattle breeds and both taurine and indicine non-grey cattle breeds in order to find genomic regions potentially related to the grey colouration. On the basis of three main drawn settings, built in order to control both the effect of the sample size and the genetic structure, we have identified some signals common to those obtained in a previous work employing only taurine cattle. In particular, using the top 1% F_st_ approach, we detected a candidate region (22.6–23.8 megabases) on chromosome 14 in which genes related to pigmentation have been already documented. In addition, when we constructed a phylogenetic tree using the significant markers identified in this study and including also the genotyping data at these loci of both the grey taurine and the extinct wild auroch, we found a topological repartition consistent with breed colour pattern rather than with the known bovine evolutionary history. Thus, on the basis of this evidence, together with the geographical distribution of the current taurine grey cattle, an ancestral indicine origin for the grey phenotype would seem to be a conceivable interpretation. In this context, a higher thermo-tolerance and less UV-induced damage of the grey phenotype might have favoured the retention of advantageous genes into the taurine genome during the post-Neolithic human-mediated cattle expansions.

## 1. Introduction

The great majority of coat colours in mammals are shades of grey and brown as a consequence of both cryptism and concealment [1]. However, coat colour in mammals has also a relevant role for other important processes such as physiological processes (UV- and thermo-tolerance), sexual selection (mate choice) and signalling, both to conspecifics and predators [2,3].

In domestic animals, strong artificial selection has led to a wide range of coat colours, where grey and brown pigmentation still remain dominant. Up to now, the genetic mechanisms of pigmentation in mammals are still largely unknown due to the complex inheritance, consisting of more than 150 genes related to the synthesis, distribution and density of eumelanin and pheomelanin pigments and its age-dependent expression [4,5]. In livestock species, a small number of genes have been recognised as being responsible for the grey coat colour. For example, in horses, a duplication of the *STX17* gene has been shown to cause a different life cycle in the hair follicle and melanocytes which promotes gradual whitening. However, such a recessive genotype has been also shown to be more prone to the incidence of melanoma, suggesting complex pleiotropic effects of genes involved in the grey coloration [6,7,8]. In contrast, dogs show a progressive greying associated with the *D* locus in a wide spectrum of dog breeds, however this phenotype does not seem to be related with pleiotropic effects increasing the incidence of melanoma [9].

In cattle, coat colour is controlled by few known genes and their corresponding alleles, such as: *ASIP* (agouti), *TYR* (albinism), *TYRP1* (brown), *KIT* (colour-sided, dominant white), *KITLG* (roan), *PMEL* (dilution), *MC1R* (extension) and *MITF* (white spotting) [10,11,12]. While several studies have highlighted a number of additional genes associated with coat colour and pattern in this species [13,14,15,16,17], less is known about the molecular basis of the grey coloration. Coat colour in cattle represents an important phenotypic trait which is considered to be a hallmark characteristic of many breeds, therefore, representing a relevant component for breeding strategies. Among cattle breeds, the grey phenotype is rather common in many taurine breeds such as those belonging to the so-called Steppe/Podolian trunk, but also in many indicine breeds. Interestingly, all these grey cattle breeds display a fawn calf which switches to grey during the first year of life. A recent study using medium density SNP arrays and adopting a multi-cohort F_st_-outlier approach has suggested several potential candidate genes when contrasting taurine grey cattle with taurine cattle displaying a non-grey phenotype [17]. In particular, 17 genes have been detected to be significantly associated in all the considered comparisons [17], suggesting the complex nature of the grey phenotype in cattle. However, whether the evolution of the grey phenotypes (in the two cattle subspecies) has been more shaped by the same pool of genes or alternatively, by converging artificial selection strategies is an attractive issue that still needs answers.

In the wave of the aforementioned study, here we expanded the above methodology to indicine grey breeds with the aim of validating the previously detected candidate genes associated with the grey phenotype and/or to identify new putative ones. The evolutionary context of *Bos taurus* makes the general framework particularly appealing, since all extant indicine and taurine breeds belong to *Bos taurus taurus* and *Bos taurus indicus*, respectively, whose ancestors diverged approximately 250 KYA [18,19]. Thus, they experienced adaptation to different environments and later were independently domesticated in the Fertile Crescent and in the Indù Valley, respectively. We performed an F_st-_outlier analysis contrasting seven indicine cattle breeds with four taurine breeds displaying a non-grey phenotype using either single comparisons or using breeds as meta-populations. In addition, we also compared the seven grey indicine breeds with the non-grey Sahiwal indicine breed in order to mitigate a possible impact of the genetic differentiation between taurine and indicine breeds. The present work provides new insight into the comprehension of the molecular mechanisms underlying coat colour in cattle.

## 2. Materials and Methods

### 2.1. Sampling and Data Filtering

A total of 62 individuals belonging to seven indicine breeds and 112 individuals belonging to four taurine cattle breeds were used in this study (detailed information about sample sizes are reported in Table 1; for details about cattle sampling and genotyping, please refer to the papers by Senczuk et al. [17] and Decker et al. [20]). All the selected indicine breeds shared phenotypic features such as the grey coat colour pattern and the calf displaying a fawn coloration from birth to the first eight months of age. Genotypic data of indicine breeds were available from a study of Decker et al. [20]. Moreover, the “non-grey” breeds selected for the pairwise contrasts with the grey ones were the same already used in a study of Senczuk et al. [17], showing the following patterns: solid black (Angus), solid white (Charolais), solid red (Limousin) and black and white piebald (Holstein).

All individuals were previously genotyped using the Illumina BovineSNP50 BeadChip and the raw SNPs were merged and filtered for minor allele frequency (--maf 0.05) and missing genotype call rate (--geno 0.1) using the software PLINK [21]. SNPs were mapped using the reference genome assembly ARS-UCD1.2 [22].

### 2.2. F_st_ Outlier Analysis

A multi-comparison approach has been used here to infer possible outlier loci using the Bayesian algorithm as implemented in the software BayeScan, and by using the default parameters [23]. This method assumes that gene frequency can be approximated by a multinomial Dirichlet distribution, thus not considering the evolutionary non-independence among samples. For this purpose, we generated three different settings to contrast grey and non-grey cattle (Table 2). Under the first scenario, we pairwise contrasted the seven grey indicine cattle with the four taurine non-grey reference breeds (Angus, Charolais, Limousin and Holstein). In the second scenario, we generated a meta-population for the indicine breeds to be pairwise contrasted with the four non-grey taurine breeds. This setting was constructed as a replicate in order to control for possible factors affecting SNPs’ outlier detection due to the limited sample size of some indicine breeds. After merging the seven indicine breeds as a single meta-population, we performed a Multi-Dimensional Scaling (MDS) analysis to check for the presence of outliers. Animals belonging to the Hariana breed resulted to be highly differentiated from the other indicine breeds (Supplementary Figure S1) and were hence discarded from this analysis. Next, under the third scenario, we contrasted the seven indicine “grey” cattle breeds (Bhagnari, Dajal, Kankrej, Tharparkar, Guzerat, Hariana and Hissar) each against an indicine “not-grey” breed (Sahiwal) as a reference. The genotypes of the Sahiwal breed have been retrieved from the dataset used in a work of Decker et al. [20]. This setting was designed in order to reduce the genetic differentiation among contrasts, reducing the impact of possible bias related to the genetic structure.

Concerning the outlier detection, we used two different approaches to retain SNPs putatively under selection. We kept loci displaying either a q-value < 0.05 or falling within the top 1% distribution of the F_st_ values. Thus, all loci passing these criteria in multiple comparisons were retained to investigate the presence of relevant genes in neighbouring (±250 kilobases) regions. We recall here that the q-value of a given locus is the minimum false discovery rate, i.e., the proportion of false positives expected among outlier markers, at which this locus may become significant. The choice to adopt, in this study, the same method for selection signatures detection as in Senczuk et al. [17] (an F_st-_outlier approach) was prompted by the need of assuring the comparability between our results and the previously published results by Senczuk et al. [17], thus allowing for a less biased signal validation.

### 2.3. Neighbor-Net Analysis

In order to check whether the topology of the detected candidate loci was consistent with colour variation, we constructed a neighbor-net graph, including all indicine and taurine breeds, based on the Reynolds genetic distances and using the eleven most significant SNPs (BTB-01532239, BTB-01530788, BTB-00557532, Hapmap46986-BTA-34282, Hapmap46735-BTA-86653, ARS-BFGL-NGS-36089, Hapmap30932-BTC-011225, Hapmap27934-BTC-065223, ARS-BFGL-NGS-11271, Hapmap48464-BTA-77646, Hapmap54435-rs29022514) that we found to be common in this study and the one by Senczuk et al. [17]. Moreover, we also included the genotypes of one extinct wild auroch, extracted from the work of Upadhyay et al. [24], and merged it to our dataset prior to neighbor-net analysis. Genetic distances and the neighbor-net graph have been obtained using the R package adegenet [25].

## 3. Results

### 3.1. Differential Selection Signatures in the Indicine “Grey” Cattle

After filtering for missing call rates and minor allele frequencies we obtained a final dataset consisting of 23,313 SNPs.

Considering the first scenario, no SNP reached the significant q-values (<0.05), except for nine loci in the Tharparkar vs. Limousin contrast (on *Bos taurus* chromosomes (BTA) 2, 5, 10, 11, 14, 19, 21 and 26), highlighted in red in Supplementary Table S1. Among these, two markers (Hapmap49624-BTA-47893 on BTA2 and the Hapmap46986-BTA-34282 on BTA14) had been previously detected as being significant in the comparison between taurine “grey” cattle and Limousin [17].

On the other hand, when we pairwise contrasted the indicine breeds as a meta-population with our taurine reference breeds, we found a total of 32 significant loci (q-value < 0.05), highlighted in red in Supplementary Table S2. Among these, only three loci (Hapmap49624-BTA-47893 on BTA2, BTB-01692321 on BTA19 and ARS-BFGL-NGS-6192 on BTA21) of the nine significant ones previously detected in the Tharparkar vs. Limousin contrast (Supplementary Table S1) were also found to be significant when the meta-population was contrasted with Limousin. Moreover, two loci were shared as significant in three meta-population pairwise comparisons (ARS-BFGL-NGS-6104 on BTA11 in Angus, Holstein and Limousin; ARS-BFGL-NGS-6192 on BTA21 in Angus, Charolais and Limousin) (Supplementary Table S2).

Next, we contrasted the seven indicine “grey” cattle breeds (Bhagnari, Dajal, Kankrej, Tharparkar, Guzerat, Hariana and Hissar) each against an indicine “not-grey” breed (Sahiwal) as reference (scenario 3, Table 2). Out of the seven pairwise comparisons, four, involving Bhagnari, Dajal, Kankrej and Tharparkar, gave significant results (q-value < 0.05) (in bold, Supplementary Table S3). Interestingly, all of them showed the same results, i.e., two consecutive SNPs in BTA6 (Hapmap48464-BTA-77646 and Hapmap54435-rs29022514). These two loci also displayed the lowest (though not significant) q-values in the other three breeds (Guzerat, Hariana and Hissar). When ranking the above BayeScan results by F_st_ and retaining as significant only the SNPs falling in the top 1% distribution, the two SNPs previously mentioned also displayed, in all the seven tested breeds, the highest F_st_ values, while none of the remaining loci falling in the top 1% distribution were observed in all the seven comparisons. Moreover, the top F_st_ loci also included, for each corresponding scenario, all the loci that displayed a significant q-value in the meta-population (Supplementary Tables S2 and S3).

Based on the good reproducibility of results obtained in the above settings using both the q-value and the top 1% SNPs parameters, we then adopted the latter approach in the contrasts involving the seven indicine “grey” cattle breeds and the four taurine reference cattle breeds. Several regions were shared by at least four indicine breeds in at least three out of the four reference breeds (Table 3, Scenario 1; Supplementary Table S1). In addition, similarly to what was previously observed in taurine “grey” cattle [17], the locus BTB-00557532 on chromosome 14 displayed significant results (falling in the top 1% F_st_ SNPs) in all the four tested scenarios (Angus, Holstein, Charolais and Limousin), notably for four, six, six and six “grey” indicine breeds, respectively. Also, the two upstream neighbouring loci (BTB-01532239 and BTB-01530788) were significant in two (Charolais and Holstein) and three (Charolais, Holstein and Limousin) scenarios, respectively. Finally, when adopting the same approach in the meta-population, all the loci identified in the taurine “grey” cattle [17], fell within the top 1% SNPs values, with several loci observed in at least two comparisons (Table 3, Scenario 2; Supplementary Table S2).

### 3.2. Neighbor-Net Graph

The neighbor-net graph based on Reynolds distances (Supplementary Figure S2) showed the presence of three principal clusters, one including all the non-grey indicine and taurine cattle except for the Gascon, the second including all the grey taurine cattle breeds and a third group composed of all the grey indicine cattle breeds. In this phylogenetic reconstruction, the wild auroch fell within the group of the non-grey cattle breeds.

## 4. Discussion

In a previous study [17], we investigated outlier loci related to coat colour by adopting a robust multi-cohort approach based on pairwise contrasts between grey and non-grey taurine cattle breeds. The most supported signals were detected on BTA2 (Hapmap49624-BTA-47893), BTA4 (Hapmap53144-ss46525999), BTA14 (with nine significant SNP loci, whose positions ranged from 22781305 to 25472332 bp) and BTA26 (ARS-BFGL-NGS-11271). Most of the genes identified in the corresponding regions have been shown to have functions at different stages of the melanogenic process (melanocyte development, melanogenesis, and pigment trafficking/transfer), suggesting the complex nature of the molecular basis of coat colour [17]. For a detailed literature analysis about the effects on coat colour by the candidate genes detected in [17], please see the Supplementary Materials Files S1–S5 from the paper by Senczuk et al. [17]. In this study, we adopted an analogous multi-cohort approach, applied to seven indicine grey cattle breeds. These were firstly pairwise contrasted against each of the four non-grey cattle breeds previously considered by Senczuk et al. [17]. Very few significant (q-value < 0.05) loci were detected, and only within one of the twenty-eight pairwise contrasts. This unexpected result prompted us to consider (i) the limited sample size of indicine breeds and (ii) the genetic divergence among taurine and indicine cattle breeds as being possible influencing factors. Indeed, when we pooled the samples belonging to single indicine breeds into a meta-population and contrasted it against each of the same four non-grey cattle breeds mentioned above, the number of loci detected as significant (q-value < 0.05) remarkably increased. Among the 32 significant outlier SNPs, two loci (ARS-BFGL-NGS-6104 and ARS-BFGL-NGS-6192) were detected as significant in three meta-population pairwise comparisons and fell within the genes *TMEM8C* and *PML*, respectively. While the *TMEM8* gene does not seem to have any role in the melanogenetic processes being involved in muscle development, the PML gene is notoriously known to cause a dilution in pigmentation in several cattle breeds [15,26,27].

Finally in the third scenario, we repeated the analysis, contrasting each of the seven indicine grey cattle breeds with a single non-grey cattle breed (Saiwhal) of indicine origin. This setting was built to avoid false positive outliers due to the increased F_st_ variance as a consequence of contrasting breeds with high genetic differentiation and to find possible convergent signals between taurine and indicine breeds. Within this scenario, the results appeared to be consistent among contrasts, as well as between the two criteria adopted for considering loci under significant differential selection (q-value < 0.05, and the top 1% SNP loci based on F_st_ ranking), supporting our initial hypothesis that both a reduced sample size and the strong genetic divergence (i.e., high F_st_ values at neutral SNP loci in the genome) between taurine and indicine breeds may have remarkably reduced the statistical power in the first scenario. In particular, two consecutive SNPs in BTA6 (Hapmap48464-BTA-77646 and Hapmap54435-rs29022514) showed both significant q-values and the highest F_st_ values, falling within a region which has been previously shown to contain genes related to carcass traits, fatty acid, lipid metabolism, milk fat secretion, eye area pigmentation and the immune system [28]. However, when adopting the alternative criterion for identifying loci under significant differential selection, the strongly supported signal, detected on BTA14 by Senczuk et al. [17] in taurine cattle breeds, was also observed for the majority of indicine breeds in all the four tested scenarios (Angus, Holstein, Charolais and Limousin). In this region, eleven loci are mapped (*XKR4*, *TRNAT-AGU*, *TMEM68*, *TGS1*, *LYN*, *CHCHD7*, *SDR16C5*, *SDR16C6*, *PENK*, *LOC112449660*, *IMPAD1*; Table 3), whose functions are presented in detail in Supplementary Table S4, together with the corresponding relevant literature. Notably, some of them (*XKR4*, *TMEM68* and *SDR16C5*) were found in a candidate region for differential selection detected by contrasting three Korean cattle breeds displaying different coat colour phenotypes [29]. In addition, *XKR4*, *TMEM68*, *TGS1*, *LYN*, *CHCHD7*, *SDR16C5* and *PENK* were found in several selection signatures in cattle with various indicine introgression levels [30,31,32,33,34,35,36,37,38].

Among the loci mapped in the candidate region of BTA14, the most interesting one appears to be the *LYN* gene, encoding a non-receptor tyrosine protein kinase. *LYN* belongs to the Src family tyrosine kinases (SFK) which are known to be involved in the modulation and regulation of many different processes such as proliferation, differentiation, migration, metabolism and apoptosis, responses to DNA damage and genotoxic agents. Interestingly, *LYN* has been shown to play a role in the Wnt/β-catenin and in the NF-kappa B signaling pathways [39,40,41], which are well known to drive early developmental events (migration and differentiation of melanocyte precursors) that may play a role in pattern formation during neural crest cell migration. Moreover, *LYN* has also been shown to interact with and induce the autophosphorylation of *KIT*, and to play a role in the control of *KIT* expression and intracellular localisation [42], with the *KIT* signalling being well known to be involved in the regulation of melanogenesis. Finally, *LYN* has been shown to be a key mediator of the effect of oestrogens (at least on osteoclastogenesis) [43] and to be involved in the regulation of androgen receptor stability and transcriptional activity [44], which could possibly give a clue on the known sexual dimorphism in the coat colour of the considered grey cattle breeds.

Another interesting candidate gene is *SDR16C5*, which encodes a member of the short-chain alcohol dehydrogenase/reductase superfamily of proteins and is involved in the oxidation of retinol to retinaldehyde, one of the steps necessary for conversion of retinol into retinoic acid, the major bioactive form of vitamin A, which influences a broad spectrum of physiological processes during embryogenesis and adulthood, regulating the expression of over 500 genes. In particular, retinoic acid has been shown to be involved in the differentiation, proliferation and maturation of melanocytes, also inducing ectopic melanocyte stem cell differentiation in the hair follicle niche [45].

*PENK* encodes a protein that is processed to generate endogenous opioid peptides (met-enkephalin and leu-enkephalin). Activation of opioid receptors in the skin by opioid peptides can regulate keratinocyte and melanocyte activities. In addition, endogenous opioids can modulate, in the skin, effects due to environmental stresses such as the solar radiation. Furthermore, *PENK* has been detected in sexually dimorphic hypothalamic regions [46] and it has been detected as being strongly differentially expressed in a whole-genome gene expression study that compared prepubertal and postpubertal Brangus heifers (a *Bos indicus*-influenced composite cattle breed) [34].

All the above evidences is in strong agreement with the features of the grey phenotype in the considered cattle breeds, such as the presence of early developmental events responsible for the formation of a pale brown ventral pattern, an altered modulation of the melanogenesis explaining the premature hair graying of the fawn coat colour observed in calves, and the appearance of a sexually dimorphic coat colour after puberty, likely as a response to steroid hormones, characterised by the onset of eumelanic pigment production, mainly in males, and, possibly through recruiting pools of melanocyte stem cells from the hair follicle niche [17]. Future studies on this topic, integrating “omic” data at different layers of biological complexity, may significantly help further elucidate the molecular mechanisms affecting premature hair greying in mammals.

Concerning the choice of the statistical methods to identify outliers, it has been shown that the ranking approach based on loci that show extreme values in terms of F_st_ could be a reasonable approach especially in all those cases in which we do not have a priori information about which loci are under selection or not, as in our case study [47]. This approach has been used in several earlier publications [48,49,50] and, while suffering from a lower incidence of Type 2 errors (false negatives), it is known to be associated with a rather high level of false positives (Type 1 error). However, it must be emphasised that the adoption of a multi-cohort approach, such as in this study, greatly contributes to reducing this risk.

The neighbor-net graph constructed using the most significant SNPs (Table 3) showed a topology consistent with the colour phenotype rather than with the phylogenetic history. Indeed, we found two principal clusters comprising all the grey cattle, one including taurine breeds and the other including the indicine breeds (Supplementary Figure S2). On the other hand, all the non-grey breeds, either indicine or taurine, clustered together with the exception of the Gascon breed (which shows a grey phenotype). However, it should be noted that the close relationship between Gascon and Limousin has been highlighted also in other studies [51,52] and could be related to their close geographic origin together with recent selection strategies to improve meat traits in Gascon, which is a dual-purpose breed. Finally, it is not surprising that the wild auroch genotype fell within the cluster of the non-grey cattle, as historical documents and depictions would indicate a deep blackish-brown fur colour pattern [53].

These results are of a certain relevance, not only because they corroborate, and thus validate, the previously published differential selection signals (likely related with the genetic architecture of premature hair greying) detected in taurine cattle [17] in an independent set of indicine cattle breeds, but also because they may point to a common origin of the genetic mechanisms underlying this adaptation-related phenotype in both cattle subspecies. A number of recent papers [24,52,54,55] reported variable proportions of indicine ancestry into central and southern Italian taurine cattle breeds belonging to the Podolian trunk (Chianina, Calvana, Romagnola, Marchigiana, Maremmana, Italian Podolica), assuming this indicine heritage in taurine cattle as remnant of remote post-domestication introgression events likely occurred in Asia and/or at the Asia/Europe border, coupled with subsequent westward cattle migration events. Under such assumption, we can hence consider that the premature hair greying observed today in cattle breeds from both subspecies was an ancestral condition in the early genetic stock of Asiatic indicine origin, where it may have played a positive role on environmental robustness (i.e., higher thermo-tolerance and lower UV-induced damage due to higher solar reflectance), while having been a posterior acquisition in taurine cattle breeds. Similar to what we observed in our study, Barbato et al. [54] also highlighted genomic regions under positive selection that have been introgressed from indicine into taurine cattle, harbouring genes with functions related to adaptive and performance traits (body size and feed efficiency). Such studies contribute to elucidating the key role of adaptive introgression in shaping the phenotypic features of modern cattle, and further our knowledge on genomic targets for improved cattle performance and resilience.

## Figures and Tables

**Table 1:** List of breeds used in this study including the subspecies, the geographic origin ^1^ and the number of samples.

Breeds	Sub-Species	Geographic Origin ^1^	Number of Samples
Bhagnari	*B. taurus indicus*	Pakistan	9
Dajal	*B. taurus indicus*	Pakistan	9
Guzerat	*B. taurus indicus*	India	3
Hariana	*B. taurus indicus*	India	10
Hissar	*B. taurus indicus*	Pakistan	9
Kankrej	*B. taurus indicus*	India	10
Tharparkar	*B. taurus indicus*	Pakistan	12
Sahiwal	*B. taurus indicus*	Pakistan	10
Angus	*B. taurus taurus*	United Kingdom	20
Holstein	*B. taurus taurus*	Holland	24
Charolais	*B. taurus taurus*	France	33
Limousin	*B. taurus taurus*	France	35

^1^ Geographic origin defined as in [20].

**Table 2:** Outline of the experimental design for each scenario.

Scenario 1	Scenario 2	Scenario 3	Reference Breeds for Scenarios 1 and 2				Reference Breed for Scenario 3
Bhagnari	Meta-population of 52 animals from six ^1^ zebuine breeds	Bhagnari	Angus	Holstein	Charolais	Limousin	Sahiwal
Dajal		Dajal					
Guzerat		Guzerat					
Hariana		Hariana					
Hissar		Hissar					
Kankrej		Kankrej					
Tharparkar		Tharparkar					

^1^ The samples belonging to the breed Hariana were removed since behaving as outliers in a MDS analysis (see main text for details).

**Table 3:** List of outlier SNPs shared between taurine breeds [17] and indicine breeds [this study].

Breed	BTA	SNP Name	Scenario 1	Scenario 2	SNP Position (Base Pairs)	Gene(s) in the ±250 Kilobases Interval
			(Indicine Breeds with Significant Results)	(Reference Breeds with Significant Results)		
ANGUS	1	ARS-BFGL-NGS-36767	7	Angus	11,400,789	*LOC112448280, LOC783459*
	8	Hapmap52255-rs29014938	6	Angus	18,589,836	*TUSC1*
	11	ARS-BFGL-NGS-36923	6	Angus	47,096,563	*LOC112448875, PSD4, PAX8, LOC100335748, LOC112448784, LOC100294952*
	14	BTB-00557532	4	Angus, Holstein, Charolais, Limousin	22,986,080	*XKR4, TMEM68, TGS1, LYN*
	18	UA-IFASA-9215	5	Angus	46,454,259	*GAPDHS, LOC100847573, TMEM147, ATP4A, LOC786928, LOC112442198, LOC101905802, LOC100140566, LOC787014, HAUS5, RBM42, ETV2, COX6B1, TRNAT-UGU, UPK1A, LOC112442195, ZBTB32, KMT2B, IGFLR1, U2AF1L4, PSENEN, LIN37, HSPB6, PROSER3, ARHGAP33, LOC101904550, PRODH2, NPHS1, LOC112442466, KIRREL2, APLP1, NFKBID, HCST, TYROBP, LRFN3, SDHAF1, SYNE4, ALKBH6, LOC107131424, CLIP3, THAP8*
HOLSTEIN	4	Hapmap53144-ss46525999	7	Holstein	76,874,783	*MYO1G, LOC112446527, PURB, MIR4657, H2AFV, PPIA, ZMIZ2, LOC112446406, OGDH, TMED4, DDX56, NPC1L1, NUDCD3, LOC104972146, CAMK2B, YKT6*
	8	Hapmap40677-BTA-121871	6	Holstein	106,052,345	*ASTN2, TRIM32*
	14	BTB-01532239	4	Charolais	22,781,305	*XKR4, TRNAT-AGU*
	14	BTB-01530788	6	Holstein, Charolais, Limousin	22,867,321	*TRNAT-AGU, XKR4, TMEM68*
	14	BTB-00557532	6	Angus, Holstein, Charolais, Limousin	22,986,080	*XKR4, TRNAT-AGU, TMEM68, TGS1, LYN*
CHAROLAIS	5	Hapmap60668-rs29018280	7	Charolais	57,356,420	*DGKA, RNF41, TRNAS-CGA, SMARCC2, MYL6, MYL6B, LOC112446711, ESYT1, LOC101903253, ZC3H10, PA2G4, ERBB3 RPS26, IKZF4, SUOX, RAB5B, CDK2, PMEL, PYM1, LOC107132498, MMP19, LOC507581, DNAJC14, ORMDL2, SARNP, GDF11, CD63, RDH5, BLOC1S1, ITGA7, METTL7B*
	5	ARS-BFGL-NGS-103394	7	Charolais	57,651,372	*LOC112446805, LOC107132498, MMP19, LOC507581, DNAJC14, ORMDL2, SARNP, GDF11, CD63, RDH5, BLOC1S1, ITGA7, METTL7B, LOC520938, LOC112446881, OR10P1, OR6C4, LOC781363, OR2AP1, LOC101903758, LOC100847112, LOC615284, LOC618816, LOC506139, LOC526713, LOC782840, LOC510046, LOC112446806, LOC529303, LOC782659*
	14	BTB-01532239	6	Charolais	22,781,305	*XKR4, TRNAT-AGU*
	14	BTB-01530788	6	Holstein, Charolais, Limousin	22,867,321	*TRNAT-AGU, XKR4, TMEM68*
	14	BTB-00557532	6	Angus, Holstein, Charolais, Limousin	22,986,080	*XKR4, TRNAT-AGU, TMEM68, TGS1, LYN*
LIMOUSIN	2	Hapmap49624-BTA-47893	7	Limousin	6,760,630	*PMS1, ORMDL1, OSGEPL1, AKAR, ASNSD1, SLC40A1, LOC100848294, WDR75*
	2	ARS-BFGL-NGS-11319	5	Limousin	6,823,052	*ORMDL1, OSGEPL1, ANKAR, ASNSD1, SLC40A1, LOC100848294, LOC101906294, WDR75, LOC101905730*
	14	BTB-01530788	4	Holstein, Charolais, Limousin	22,867,321	*TRNAT-AGU, XKR4, TMEM68*
	14	BTB-00557532	6	Angus, Holstein, Charolais, Limousin	22,986,080	*XKR4, TRNAT-AGU, TMEM68, TGS1, LYN*
	14	BTB-00559128	5	Limousin	23,539,175	*LOC112449630, MOS, PLAG1, CHCHD7, SDR16C5, SDR16C6, PENK*
	14	Hapmap46986-BTA-34282	7	Angus, Holstein, Charolais, Limousin	23,630,896	*CHCHD7 SDR16C5 SDR16C6 PENK LOC112449660 IMPAD1*
	14	Hapmap46735-BTA-86653	4	Limousin	23,725,488	*SDR16C6, PENK, LOC112449660, IMPAD1*

Number of chromosomes (BTA), SNP names and positions are reported, together with number of indicine grey breeds displaying those loci as significant, in at least 4 pairwise comparisons, in scenario 1 (see Supplementary Table S1), and the taurine non-grey reference cattle breeds displaying significant results when contrasted to the indicine meta-population in scenario 2 (see Supplementary Table S2). In both cases, the significance criterion was based on the top 1% F_st_ approach. For direct comparison of results in this study and in [17], please, refer to the Supplementary Table S2 of the paper published by Senczuk et al., 2020 [17]. Genes are highlighted in bold when the SNP locus falls within the gene boundaries.

## Data Availability

Not applicable.

## Supplementary Materials

The following supporting information can be downloaded at: https://www.mdpi.com/article/10.3390/genes13091601/s1, Figure S1: Multidimensional Scaling (MDS) analysis of the seven indicine breeds; Figure S2: Neighbor-net graph constructed using the most significant SNPs; Table S1: Signatures of selection observed contrasting indicine breeds vs. taurine breeds; Table S2: Signatures of selection observed contrasting a meta-population of the seven indicine breeds vs. each taurine breed; Table S3: Signatures of selection observed contrasting the seven indicine breeds vs. Sahiwal. Table S4: Functions of genes in the candidate region in BTA14.
