# Supplementary figures and images for: Seven Shades of Grey: A Follow-Up Study on the Molecular Basis of Coat Colour in Indicine Grey Cattle Using Genome-Wide SNP Data
Source: Genes (Basel). 2022 Sep 7;13(9):1601. doi: 10.3390/genes13091601 (PMC9498432; doi:10.3390/genes13091601)

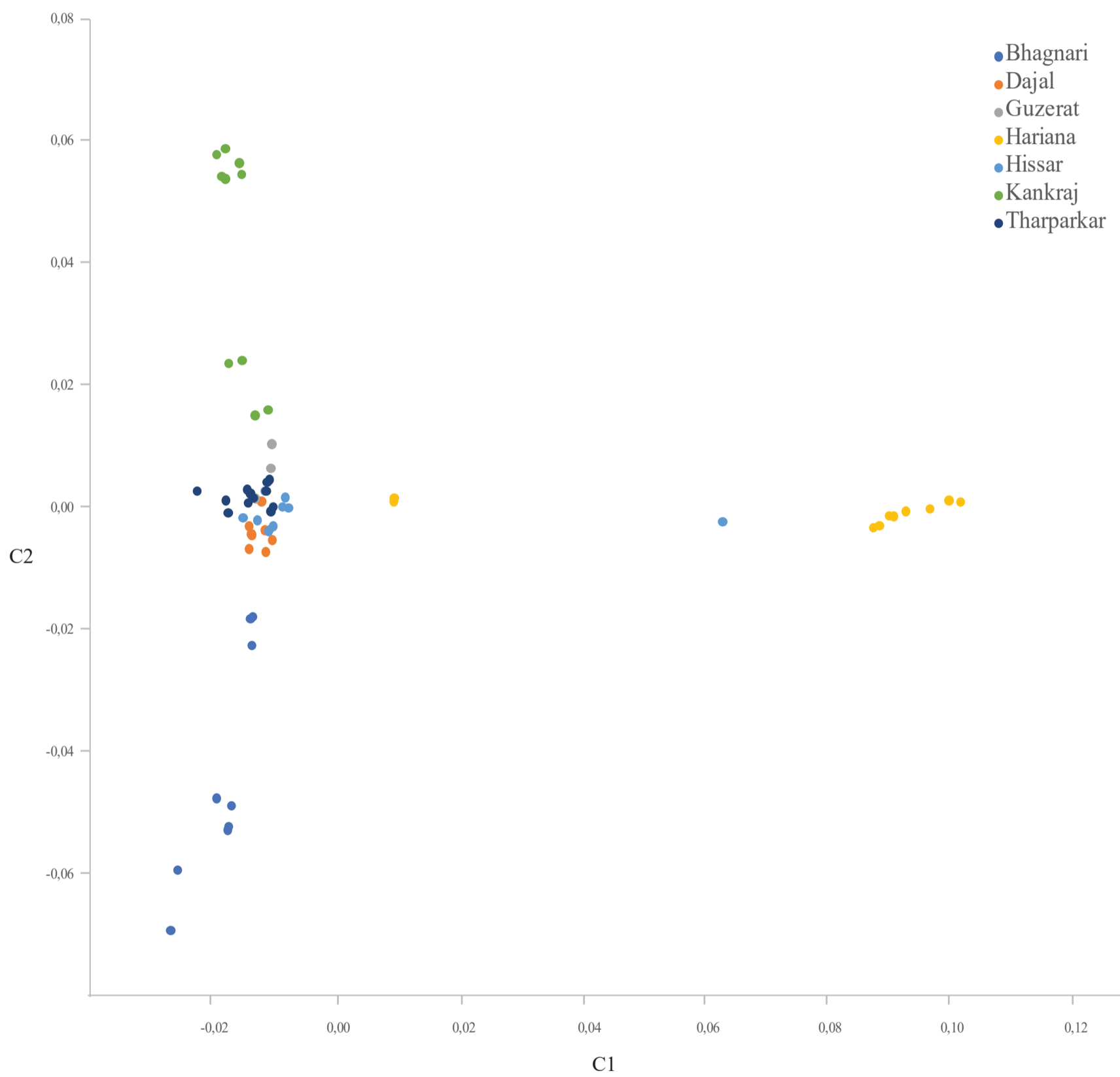

Supplement: Supplementary file 1 [file genes-13-01601-s001.zip › genes-1766571-supplementary/Supplementary material/Supplementary Figure S1.pdf]

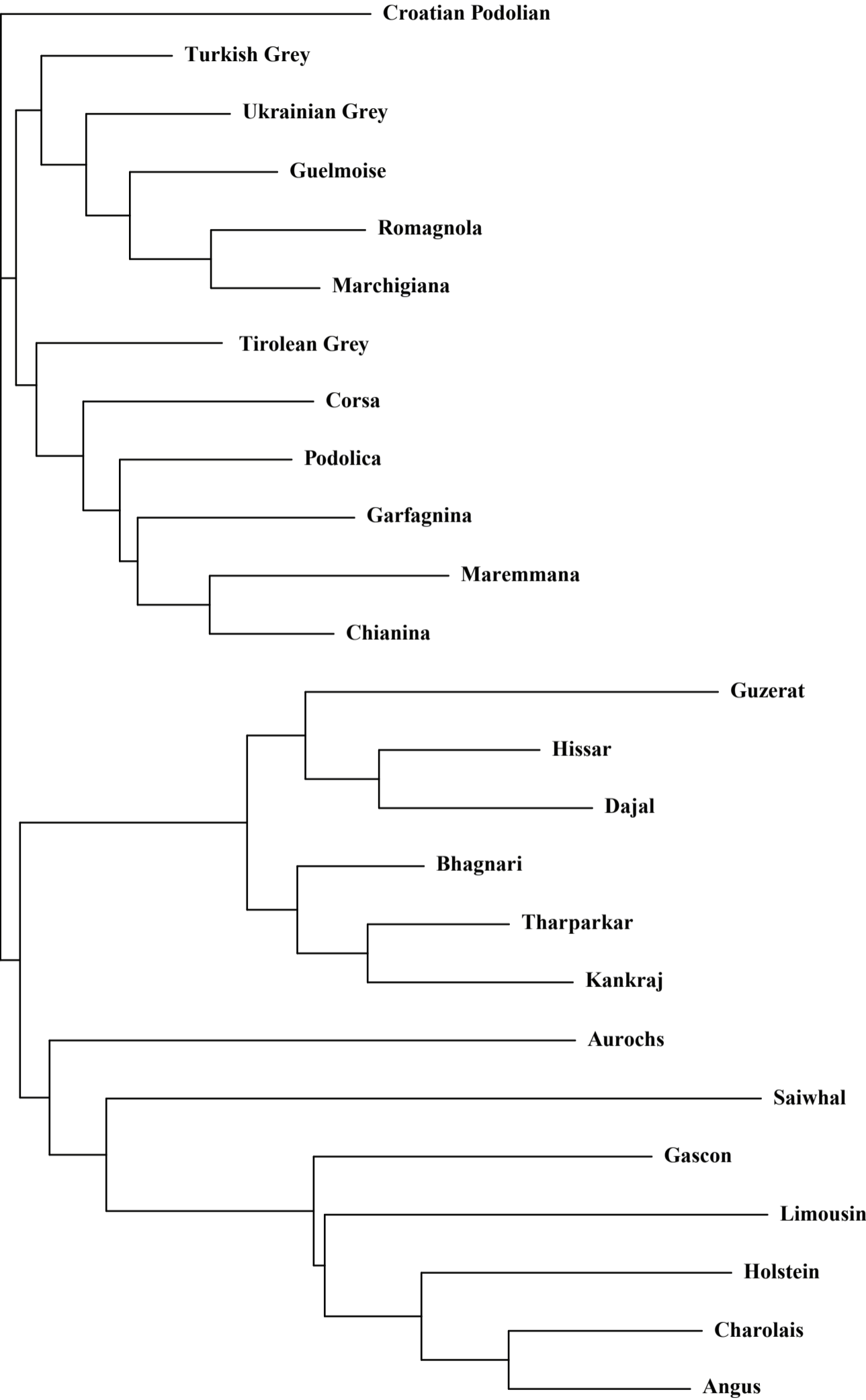

Supplement: Supplementary file 1 [file genes-13-01601-s001.zip › genes-1766571-supplementary/Supplementary material/Supplementary Figure S2.pdf]
